# CRISPR/Cas9‐Mediated Gene Knockout Reveals a Nonredundant Role for p16^INK4A^ in Controlling TCR‐Dependent and Independent CD8 T Cell Expansion

**DOI:** 10.1002/eji.70224

**Published:** 2026-06-25

**Authors:** Silvia Fiori, Cecilia Adragna, Emilia Malvicini, Tommaso Basini, Donatella Galgano, Edoardo Scarpa, Sandra Jovic, Niklas A. Schmacke, Veit Hornung, Federica Sallusto, Ludovica Bruno, Antonio Lanzavecchia, Manuel Albanese

**Affiliations:** ^1^ National Institute of Molecular Genetics Milan Italy; ^2^ Department for Clinical Sciences and Community Health (DISCCO) (DoE 2023–2027) University of Milan Milan Italy; ^3^ Institute for Research in Biomedicine Bellinzona Switzerland; ^4^ Department of Pharmaceutical Sciences University of Milan Milan Italy; ^5^ Gene Center and Department of Biochemistry Ludwig‐Maximilians‐Universität München Munich Germany; ^6^ Institute of Microbiology ETH Zurich Zurich Switzerland

**Keywords:** CD8 T cells, cell cycle, CRISPR/Cas9, cytotoxicity, gene editing, p16^INK4A^, proliferation

## Abstract

The possibility of enhancing T cell function by deleting specific genes represents a long‐sought goal in preclinical studies and ultimately for clinical applications. Using CRISPR/Cas9 genome editing, we report that, in human cytotoxic CD8 T cell clones, the cell cycle checkpoint gene *CDKN2A*, encoding p16^INK4A^, plays a nonredundant role in controlling T cell receptor (TCR)‐dependent and independent cell expansion. Deletion of *CDKN2A* dramatically enhanced antigen‐driven and homeostatic proliferation, while preserving effector functions. In contrast, the deletion of other cell cycle inhibitors (*CDKN1B*, *CDKN2C*, and *CDKN2D*), alone or in combination, had no impact on T cell proliferation. We also report that mediator complex subunit 12 (MED12) and the E3 ubiquitin ligase CBL‐B deletions did not affect proliferative capacity of CD8 T cell clones. Interestingly, deletion of the negative regulator of Ras signaling, RASA2, increased antigen sensitivity and cytotoxic activity, while not improving in vitro expansion. Collectively, these findings reveal a unique and critical nonredundant role for p16^INK4A^ in regulating CD8 T cells. Deletion of *CDKN2A* offers a promising strategy to enhance CD8 T cell expansion ex vivo, thereby improving TCR discovery pipelines and, potentially, therapeutic applications.

## Graphical abstract

Ex vivo characterization of antigen‐specific CD8 T cells is crucial but often limited by expansion bottlenecks. We identify p16^INK4A^ as a key regulator of TCR‐dependent and homeostatic proliferation. CRISPR/Cas9 deletion of *CDKN2A* rescues proliferation and improves IFN‐γ secretion while maintaining TCR‐dependency, enabling robust expansion for functional characterization and clinical applications.

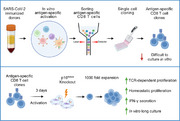

## 1 Introduction

Cytotoxic CD8 T lymphocytes are crucial for eliminating infected and neoplastic cells, forming the basis of adoptive T cell therapies. The success of such therapies depends on selecting high‐affinity T cell receptors (TCRs) and ensuring long‐term persistence of transferred T cells [1, 2, 3]. However, challenges in culturing and characterizing CD8 T cell clones, particularly from senescent or exhausted populations, limit the breadth of analyzable TCR repertoires and hinder in‐depth epitope specificity studies [4].

An exciting possibility to improve T cell proliferation and effector function is knocking out individual genes. By performing genome‐wide CRISPR knockout (KO) screens in primary human T cells, Shifrut et al. identified a few candidate genes such as *CD5*, *CBLB*, and *RASA2* [5]. In a subsequent study, they reported that *RASA2* ablation in TCR‐T cells and chimeric antigen receptor (CAR) T cells enhanced their cytolytic activity, cytokine production, and fitness in a tumor model as well as their resistance to immunosuppressive conditions [6]. In another screening, Freitas et al. reported that deletion of *MED12*, a component of the mediator kinase module, could lead to enhanced effector function and increased expansion of CAR T cells [7].

In this study, we established a pipeline to knock out up to three genes simultaneously in human antigen‐specific CD8 T cell clones using CRISPR/Cas9. Various gene KOs were assessed for their impact on T cell functions, viability, and proliferative capacity. Although we found only marginal effects from MED12, CBL‐B, and RASA2 KOs, with increased killing in the latter, we identified the CDK inhibitor p16^INK4A^ as a critical nonredundant regulator of CD8 T cell expansion. Deletion of the p16^INK4A^‐encoding gene *CDKN2A* significantly enhanced antigen‐driven and homeostatic proliferation, enabling rescue and dramatic expansion of CD8 T cell clones ex vivo.

## 2 Results

### 2.1 Phenotypic Effects of RASA2 KOs and Other Proteins on Antigen‐Specific CD8 T Cell Clones

Given the practical interest of improving T cell expansion ex vivo and in vivo, we evaluated the effect of deleting *RASA2*, *CBLB*, and *MED12* that were previously reported to enhance T cell function and proliferation [5, 6, 7], as well as genes that act as cell cycle checkpoints, such as members of the INK/KIP family [8, 9]. The experiments were performed on SARS‐CoV‐2 Spike‐specific CD8 T cell clones isolated from memory T cells of immune donors that were repeatedly stimulated by vaccination and infection (Figure S1). These clones have high sensitivity to specific peptide epitopes and can be stimulated with specific antigen in the form of Spike‐expressing autologous lymphoblastoid cell lines (LCLs) but typically possess only a limited *in vitro* expansion potential.

Using combinations of two or three gRNAs targeting the same gene, we reproducibly achieved high KO efficiencies (Figure S2). In all cases, the predominant DNA species corresponded to the larger fragment deletion generated between the gRNA target sites (Figure S2A–C), also confirmed by the presence of a smaller PCR band in KO compared to wild‐type (WT) T cells (Figure S2D). In these conditions, we achieved near complete editing efficiency in *MED12* (90.6% ± 8.5%), *CBLB* (95.2% ± 3.9%), and *RASA2* (89% ± 8.7%) (Figure S2C,D) but preserving viability of edited CD8 T cells (Figure S2E,F), which enabled successful and unbiased characterization of their phenotype.

**FIGURE 1:**
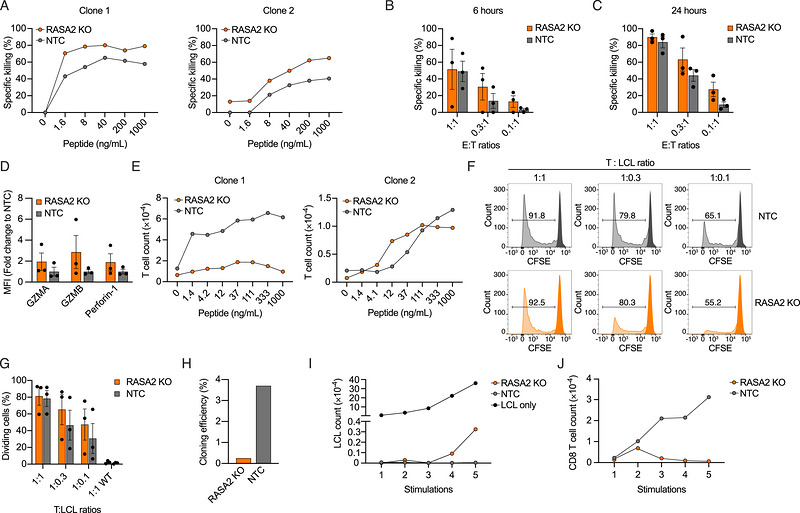
RASA2 KO enhances CD8 T cell clone antigen sensitivity and cytotoxic functions but heighten susceptibility to activation‐induced cell death (AICD). (A) CD8‐mediated cytotoxicity of NTC and RASA2 KO SARS‐CoV‐2 Spike‐specific CD8 T cell clones measured as percentage of the specific killing of autologous LCLs in the presence of different concentrations of cognate Spike peptide. Effector to target (E:T) ratio used was 1:1. LCLs killing was measured by flow cytometry after 6 h. Results from two different CD8 T cell clones deriving from two donors are shown. (B) CD8‐mediated cytotoxicity of NTC and RASA2 KO SARS‐CoV‐2 Spike‐specific CD8 T cell clones from three different donors measured as percentage of the specific killing of autologous LCLs expressing Spike S1 subunit at different E:T ratios. LCLs killing was measured by flow cytometry after 6 h (B) and 24 h (C) of incubation. Mean and s.e.m. are shown (*n* = 3). Paired *t*‐test was performed resulting in no statistical significance. (D) Intracellular expression of Granzyme A (GZMA), Granzyme B (GZMB), and Perforin‐1 measured by flow cytometry in RASA2 KO and NTC CD8 T cell clones from three different donors under resting conditions. Mean and s.e.m. of fold change relative to NTC are shown (*n* = 3). Paired *t*‐test was performed, resulting in no statistical significance. (E) Proliferation measured as absolute T cell count of NTC and RASA2 KO SARS‐CoV‐2 Spike‐specific CD8 T cell clones in response to autologous LCLs pulsed with different concentrations of Spike peptides. T:LCL ratio used was 2:1. Cell count was measured by flow cytometry 5 days after stimulation. Results from two different CD8 T cell clones deriving from two donors are shown. (F and G) Proliferation measured as CFSE dilution of NTC and RASA2 KO SARS‐CoV‐2 Spike‐specific CD8 T cell clones stimulated with autologous LCLs expressing S1 or S2 subunit, depending on the clone specificity. Proliferation was measured by flow cytometry 7 days after stimulation. Displayed are representative histograms (F) and bar plots (mean ± s.e.m., *n* = 3) (G). Paired *t*‐test was performed, resulting in no statistical significance. Dark gray and dark orange profiles show resting T cells, and light gray and orange profiles show stimulated T cells. (H) Cloning efficiency (%) obtained by sub‐cloning an antigen‐specific CD8 T cell clone from one donor in the presence of PHA and irradiated allogenic feeders (*n* = 1). (I and J) LCLs and T cells count monitored every 24 h after the addition of fresh autologous LCL‐S1 in co‐culture with a Spike‐specific CD8 T cell clone, at 1:1 T:LCL ratio. Mean of two technical replicates from one CD8 T cell clone is shown (*n* = 1). KO, knockout; LCL, lymphoblastoid cell line; NTC, non‐targeting control.

Using the above approach, we investigated the effect of *RASA2*, *MED12*, and *CBLB* deletion on key T cell functions, such as cytotoxicity, IFN‐γ production, and proliferation of Spike‐specific CD8 T cell clones stimulated by increasing peptide concentrations (Figure S3). When compared to non‐targeting control (NTC) cells, *MED12* KO T cells expressed increased levels of CD25 (Figure S3A), but their antigen‐dependent proliferation (Figure S3B,C) and specific killing activity at short time point (3 and 6 h) were reduced (Figure S3D,E). However, the recovery of killing efficacy by 24 h (Figure S3E right panel) suggests a potential reduction of the kinetic of cell‐mediated cytotoxicity rather than the overall cytotoxic capacity. Conversely, IFN‐γ production was not affected (Figure S3F). Furthermore, following *CBLB* KO, we observed minimal alterations in proliferation, cytotoxicity, and IFN‐γ production compared to NTC cells (Figure S3A,C,E,F). Interestingly, *RASA2* ablation increased antigen‐dependent cytotoxic activity in an experiment using LCL pulsed with the cognate peptide at all the different time points tested (Figure 1A and Figure S3E). To further investigate the RASA2 deletion phenotype, we stimulated NTC and KO T cells with increasing numbers of autologous LCLs expressing Spike S1 or S2 subunit, depending on clone specificity. RASA2 KO CD8 T cells demonstrated increased cytotoxic activity compared to NTC cells, evidenced by more rapid and sensitive killing of target cells, even at the lowest effector to target (E:T) ratio (Figure 1B,C). The increased cytotoxicity correlated with an increase in intracellular concentrations of Granzyme A (GZMA), Granzyme B (GZMB), and Perforin‐1 (Figure 1D and Figure S3G). The effect of *RASA2* deletion on proliferation in response to cognate peptide showed interclonal variability (Figure 1E). Consistent with this, CFSE dilution assay showed that RASA2 KO T cells proliferated modestly more than NTC cells in response to low numbers of S1/S2‐expressing LCLs but failed to accumulate in response to higher LCL numbers (Figure 1F,G). Furthermore, although NTC T cells showed a low cloning efficiency, consistent with the limited proliferation potential of differentiated antigen‐experienced T cell clones, RASA2 KO T cells exhibited severely impaired cloning efficiency (Figure 1H).

We also investigated the capacity of RASA2‐deficient and NTC T cells to maintain functionality upon continuous antigenic stimulation, a condition found in tumors and chronic infections [10, 11]. To this end, we added autologous LCLs expressing S1 subunit to a Spike S1‐specific T cell clone every 48 h for five rounds of stimulations and counted the number of surviving target cells (LCLs) 24 h after each round (Figure 1I). In contrast with NTC T cells, RASA2‐deficient T cells failed to control proliferation of S1‐expressing LCLs after the third round of stimulation. Examination of viable CD8 T cell counts in culture revealed that RASA2 KO T cells did not expand as effectively as NTC cells (Figure 1J).

These findings suggest that *RASA2* deletion increases the sensitivity of T cells to low antigen doses, consistent with the role of RASA2 as a negative regulator of signaling [6, 12]. Additionally, *RASA2* deletion enhances the cytotoxic functions of T cells but also renders them more susceptible to activation‐induced cell death (AICD) following strong and repetitive TCR stimulations, thus limiting their long‐term functionality. These findings highlight the complex role of RASA2 in regulating T cell function and survival.

Collectively, our findings differ to some extent from previous studies [5, 6, 7], suggesting that the effects of these gene KOs may depend on the nature or differentiation state of the T cells tested, although we also observed a degree of variability between clones. The complex behavior of RASA2‐deficient cells, particularly under chronic stimulation, underscores the need for careful evaluation of potential trade‐offs across different T cell phenotypes when considering genome‐editing strategies for potential T cell therapies.

### 2.2 p16^INK4A^ Is a Nonredundant Regulator of Antigen‐Driven CD8 T Cell Proliferation

Given that our main goal was to improve CD8 T cell clonal expansion, we evaluated the effect of knocking out genes that act as cell cycle checkpoints. Progression through the cell cycle is regulated by cyclins and cyclin‐dependent kinases (CDKs), whose activity is modulated by CDK inhibitors (CDKis) [13].

We analyzed the expression of INK and KIP CDKis family CDK inhibitors in total CD8 T cells from a healthy donor using single‐cell RNA sequencing (Figure S4). Interestingly, among these, only *CDKN2A* (p16^INK4A^), *CDKN2C* (p18^INK4C^), *CDKN2D* (p19^INK4D^), and *CDKN1B* (p27^KIP1^) were consistently expressed among different CD8 T cell subsets (Figure S4B,C). *CDKN2D* and *CDKN1B* showed the highest expression levels, whereas *CDKN2A* and *CDKN2C* were moderately expressed, particularly within the *MKI67* ^+^ proliferating subsets (Figure S4A–C), suggesting a potential role in the regulation of proliferation.

We therefore explored the role of CDKis in the regulation of homeostatic and antigen‐driven T cell proliferation by deleting individually the four most expressed members of the INK and KIP CDKi families [14, 15]: *CDKN2A* (p16^INK4A^), *CDKN2C* (p18^INK4C^), *CDKN2D* (p19^INK4D^), and *CDKN1B* (p27^KIP1^) (Figure S5A–F).

**FIGURE 2:**
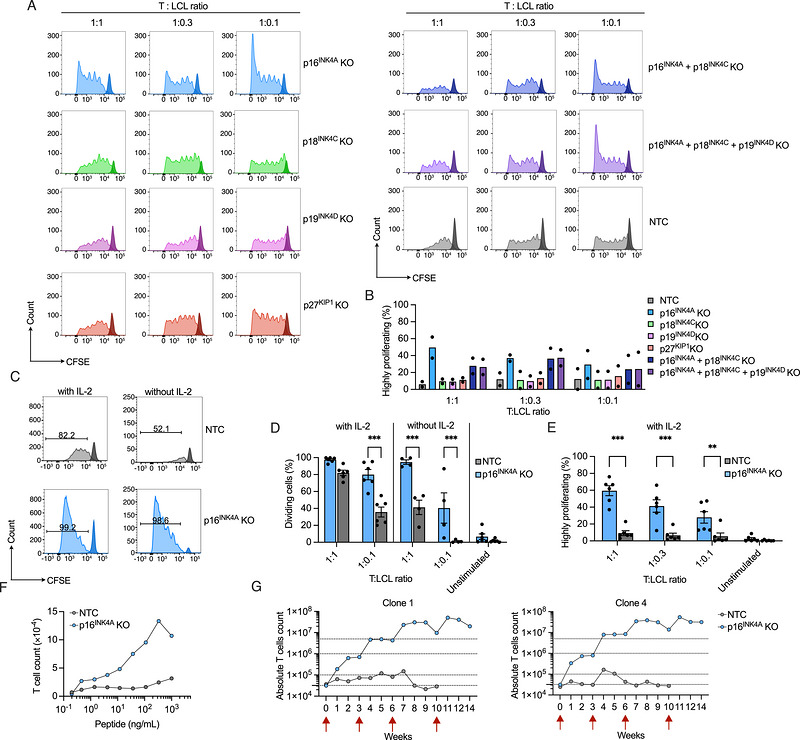
p16^INK4A^ KO confers proliferation advantage in response to antigen‐specific stimulation in antigen‐specific CD8 T cell clones. (A) Proliferation measured as CFSE dilution of a representative SARS‐CoV‐2 Spike‐specific CD8 T cell clone (*n* = 2) upon stimulation with different ratios of autologous LCLs expressing Spike S1 subunit (light colors), compared to unstimulated cells (dark colors). Shown are single p16^INK4A^, p18^INK4C^, p19^INK4D^, and p27^KIP1^ KO, double p16^INK4A^ p18^INK4C^ KO and triple p16^INK4A^ p18^INK4C^ p19^INK4D^ KO and NTC. Measurement was performed after 7 days from stimulation. (B) Bar plot showing the quantification of the fraction of highly proliferating T cells shown in A. Mean of two donors is shown (*n* = 2). (C–E) Proliferation measured as CFSE dilution of NTC and p16^INK4A^ KO SARS‐CoV‐2 Spike‐specific CD8 T cell clones from four different donors stimulated with autologous LCLs expressing Spike S1 or S2 subunit in the presence (*n* = 6) or in the absence of exogenous IL‐2 (*n* = 4). Shown are representative histograms of stimulated (light colors) and unstimulated (dark colors) cells (C) and bar plots showing the frequency of total proliferating T cells (D) and highly proliferating T cells (E). Measurement was performed by flow cytometry 7 days after stimulation. Statistics indicate significance by paired *t*‐test, *p < 0.05, **p < 0.01, and ***p < 0.001. (F) Proliferation measured as T cell count of NTC and p16^INK4A^ KO SARS‐CoV‐2 Spike S1‐specific CD8 T cell clone from one donor (*n* = 1) in response to autologous LCLs pulsed with different concentrations of cognate peptide. T:LCL ratio used was 2:1. Cell count was performed by flow cytometry 5 days after the initial stimulation. (G) Proliferation of p16^INK4A^ KO and NTC SARS‐CoV‐2 Spike‐specific CD8 T cell clones measured as T cell count. T cells were restimulated with PHA and allogeneic irradiated feeders every 3 weeks (time points indicated with red arrows in the *x* axis). Measurements were performed by flow cytometry every week. Two different clones from one donor are shown. KO, knockout; LCL, lymphoblastoid cell line; NTC, non‐targeting control.

Surprisingly, the deletion of *CDKN2A*, encoding p16^INK4A^, showed a clear phenotype resulting in increased antigen‐driven proliferation and increased sensitivity to low number of S1‐expressing LCLs (Figure 2A,B). In contrast, individual deletion of the other CDKis, including *CDKN2C*, *CDKN2D*, and *CDKN1B*, did not affect the proliferative response (Figure 2A,B). The simultaneous deletion of *CDKN2A* along with *CDKN2C* or the triple KO of *CDKN2A*, *CDKN2C*, and *CDKN2D* did not further enhance proliferation compared to deletion of *CDKN2A* alone (Figure 2A,B and Figure S5G). Interestingly, although CD8 T cell clones require exogenous IL‐2 for survival and proliferation, p16^INK4A^ KO clones initially proliferated even in the absence of IL‐2 when exposed to strong antigenic stimulation (Figure 2C,D and Figure S6A). Additionally, p16^INK4A^ KO dramatically improved the fraction of T cells undergoing multiple rounds of replications in response to the antigenic stimulation (Figure 2E and Figure S6A).

The proliferative advantage of p16^INK4A^‐deficient CD8 T cells was striking (Figure 2E,F). When stimulated with high peptide concentrations, p16^INK4A^‐deficient CD8 T cells achieved over 30‐fold expansion in only 5 days (Figure 2F). Furthermore, when re‐stimulated multiple times over 14 weeks, NTC T cell clones only reached three‐ to six‐fold expansion and showed reduced viability after the second stimulation, confirming their low survival and expansion properties in vitro, whereas p16^INK4A^‐deficient T cells achieved up to 1000‐fold expansion and maintained better viability throughout the culture period (Figure 2G).

**FIGURE 3:**
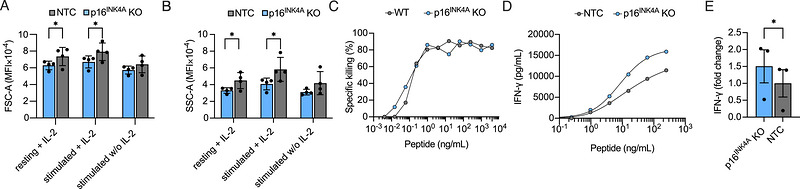
p16^INK4A^ deficient CD8 T cell clones have reduced size and preserve effector functions. (A and B) Size and granularity of p16^INK4A^ KO and NTC SARS‐CoV‐2 Spike‐specific CD8 T cell clones measured as FSC‐A and SSC‐A by flow cytometry. Cells were kept in the presence of exogenous IL‐2 only or stimulated with LCL‐S1 in the presence or in the absence of exogenous IL‐2 (n = 4). Statistics indicate significance by paired *t*‐test, *p < 0.05. (C) CD8 mediated cytotoxicity of Spike‐specific CD8 T cell clone measured as percentage of specific killing of autologous LCLs in the presence of different concentrations of cognate peptide. E:T ratio used was 1:1. Percentage of LCL killing was measured by flow cytometry after 6 h. One representative experiment out of 3 different donors is shown (*n* = 3). Replicates are shown in Figure S6D. (D) IFN‐γ production measured by ELISA of NTC and p16^INK4A^ KO Spike‐specific CD8 T cell clone upon overnight co‐coculture with autologous LCLs pulsed with titrated concentration of Spike cognate peptides. One representative experiment out of three different donors is shown (*n* = 3). Replicates are shown in Figure S6E. (E) Bar plot showing IFN‐γ release upon stimulation with LCLs pulsed with 250 ng/mL of Spike cognate peptides. Mean and s.e.m. of fold change relative to NTC are shown (*n* = 3). Statistics indicate significance by paired *t*‐test, *p < 0.05. KO, knockout; NTC, non‐targeting control; WT, wild‐type.

Phenotypic analysis showed that p16^INK4A^ KO T cells were of smaller size and exhibited reduced granular complexity compared to NTC cells, both in resting conditions and upon stimulation in the presence or absence of IL‐2 (Figure 3A,B), which was consistent overtime (Figure S6B,C). This phenotype is likely explained by reduced growth due to rapid entry into the S phase. However, despite these changes, p16^INK4A^ KO CD8 T cell clones displayed cytotoxic activity comparable to that of control clones (Figure 3C and Figure S6D) and a modest but significant increase in IFN‐γ production upon antigenic stimulation (Figure 3D,E and Figure S6E). In contrast, production of other pro‐inflammatory cytokines, such as TNF‐α and IL‐2, as well as effector molecules, including FasL, GZMA, GZMB, Perforin‐1, was not significantly altered (Figure S6F).

Collectively, these results establish p16^INK4A^ as a critical, nonredundant regulator of antigen‐driven CD8 T cell proliferation, resulting in higher sensitivity to antigenic stimulation and lower dependence on exogenous IL‐2 without affecting effector functions.

### 2.3 *CDKN2A* Deletion Facilitates Cytokine‐Driven CD8 T Cell Expansion and Confers a Proliferative Competitive Advantage

**FIGURE 4:**
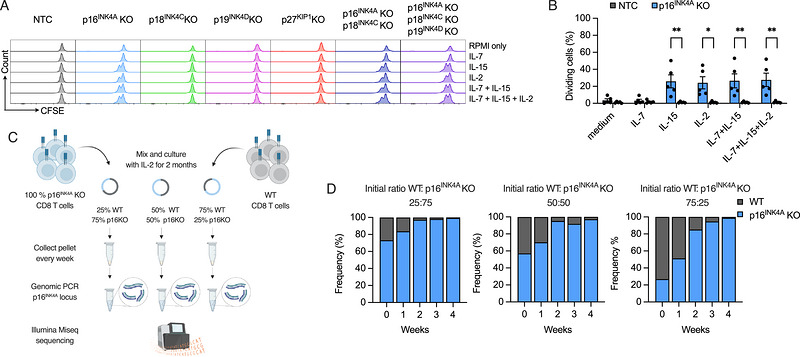
p16^INK4A^ KO confers proliferation advantage in response to homeostatic cytokine and in competitive conditions. (A) Representative histograms of CFSE dilution of one SARS‐CoV‐2 Spike S1‐specific CD8 T cell clone (*n* = 1) upon stimulation with homeostatic cytokines. Shown are NTC and p16^INK4A^, p18^INK4C^, p19^INK4D^, p27^KIP1^ KO and double p16^INK4A^, p18^INK4C^ KO, and triple p16^INK4A^, p18^INK4C^, p19^INK4D^ KO. Measurement was performed 7 days after stimulation. (B) Bar plot showing quantification of proliferation by CFSE dilution of five different NTC and p16^INK4A^ KO SARS‐CoV‐2 Spike‐specific CD8 T cell clones from 4 donors upon stimulation with homeostatic cytokines. Mean and s.e.m. are shown (*n* = 5). Statistics indicate significance by paired *t*‐test, *p < 0.05, and **p < 0.01. (C) Schematic representation of competitive proliferation experiment. WT and p16^INK4A^ KO clones were mixed in the initial ratios of 25:75, 50:50, and 75:25 (WT: p16^INK4A^ KO) and then stimulated with autologous LCL expressing Spike S1 subunit. Cell lysate was acquired in the following weeks, and PCR followed by Illumina MiSeq sequencing on the target locus was performed. Created with BioRender.com. (D) Bar plots showing the frequency of INDELs obtained from Illumina MiSeq sequencing from the lysate cells acquired at the indicated time points (*n* = 1). KO, knockout; NTC, non‐targeting control; WT, wild‐type.

To further evaluate the nonredundant function of p16^INK4A^, we measured the response of T cell clones to IL‐2, IL‐7, and IL‐15 as a surrogate of in vivo TCR‐independent homeostatic expansion [16]. Deletion of *CDKN2A* but not of the other tested CDKis significantly enhanced T cell responsiveness to IL‐2 and IL‐15, with minimal additive effects when the cytokines were combined (Figure 4A,B).

During the T cell response, specific T cell clones compete for special proximity to cytokines providing cells resulting in the selection and expansion of the most fit clones in the memory pool. To evaluate T cell competitiveness in vitro, we mixed WT and p16^INK4A^‐deficient CD8 T cells at different ratios before antigenic stimulation (Figure 4C). At different time points the prevalence of WT and targeted T cells was monitored by specific amplification of the *CDKN2A* genomic locus. As shown in Figure 4D, p16^INK4A^‐deficient T cells progressively accumulated and on Day 28 represented the absolute majority, even when present initially as 25% of total T cells (Figure 4D and Figure S7A,B).

In conclusion, our results demonstrate that *CDKN2A* deletion not only enhances the short‐term proliferation and antigen sensitivity of CD8 T cells but also improves their survival capacity under homeostatic conditions as well as their competitiveness with WT T cells of the same specificity.

## 3 Discussion and Conclusion

The possibility of enhancing T cell function represents a long‐sought goal in preclinical studies and ultimately for clinical applications. Two groundbreaking studies using CRISPR screens on human primary T cells [5, 6] or CAR T cells [7] showed that deletion of *MED12*, *CBLB*, or *RASA2* could enhance effector function and proliferation of CAR T cells [5, 6, 7]. In this study, using antigen‐specific human CD8 T cell clones, we report that MED12 or CBL‐B KO in human CD8 T cell clones have only modest effects on T cell proliferation and function, whereas RASA2 KO, despite increasing antigen sensitivity and killing activity, partially compromised T cell expansion due to AICD.

These differences may be attributed to different models of T cells used in our experiments. Although previous studies have based their models on total CD3 or CD8 T cells and CAR T cells, we focused on antigen‐specific CD8 T cell clones, recalled from donors’ CD8 T cell repertoire by antigenic stimulation ex vivo, with the aim of recovering their expansion capacity in vitro and improving the TCR discovery pipelines.

To achieve this goal, we identified cell cycle checkpoints as interesting targets. Previous study using human helper T cell clones and mouse alloreactive CD4 T cells elucidated the role of p27^KIP1^ in controlling T cell proliferation upon activation [9]. p27^KIP1^ is highly expressed in resting T cells, where it prevents cell cycle progression by interfering with the association of CDK2 to cyclin E and therefore blocking the transition into S phase. Upon productive stimulation through the TCR, CD28 co‐stimulation, and IL‐2 signaling, p27^KIP1^ undergoes ubiquitination and degradation, relieving this blockade [9, 17]. Additionally, studies in mice demonstrated that the germline deletion of *CDKN2C*, encoding p18^INK4C^, increases CDK6 association with cyclin D, promoting G1 phase progression and ultimately T cell proliferation [8]. However, in the same study, the group reported no significant enhanced proliferation in p27^KIP1^ and p19^INK4D^ deleted murine T lymphocytes, despite their documented high expression in resting T cells. This highlights an inadequate understanding of cell cycle control in T cells.

Our study provides a comprehensive evaluation of the role of four different CDKis, namely, p16^INK4A^, p18^INK4C^, p19^INK4D^, and p27^KIP1^ in CD8 T cell expansion. Our systematic analysis revealed that in human CD8 T cells only p16^INK4A^ plays a nonredundant role in restraining T cell proliferation in response to homeostatic and TCR‐based stimulation. Deletion of p16^INK4A^ significantly enhanced the sensitivity of CD8 T cells to both antigen‐driven and homeostatic proliferation, enabling over 1000‐fold expansion after long‐term culture. This result is in line with the previous observation of Migliaccio et al., who demonstrated that CD8 T cells that express p16^INK4A^ are arrested in the G0/G1 phase of the cell cycle and proposed p16^INK4A^ as a factor that controls replication potential of CD8 T cells in vitro upon mitogen stimulus, such as phytohemagglutinin (PHA) and IL‐2 [18]. Deletion of p16^INK4A^ may force the pool of cell cycle‐arrested T cells to enter the S phase, increasing the magnitude of final response. It is well established that T cells express different levels of cell cycle regulators depending on their differentiation state [19, 20, 21]. For instance, memory T cells are characterized by higher levels of active CDK6 and lower levels of p27^KIP1^ with respect to naïve T cells, reflecting a different preparedness to respond to antigenic stimulation [20]. Furthermore, evidence shows that antigen‐specific murine memory CD8 T cells are characterized by high mRNA levels of p16^INK4A^ [22]. It is therefore possible that, within the specific transcriptional state of the ex vivo isolated antigen‐specific CD8 T cells analyzed in this study, p16^INK4A^ represents a predominant factor regulating cell cycle progression.

Importantly, p16^INK4A^ KO did not impair effector functions, such as cytotoxicity and cytokine production. The ability of p16^INK4A^ KO T cells to undergo robust proliferation, while maintaining TCR dependence and effector phenotype, positions this approach as a valuable tool to overcome major hurdles in CD8 T cell research and therapy. The enhanced expansion potential could facilitate in‐depth characterization of antigen‐specific T cells and generation of larger numbers of therapeutic cells from limited starting material.

This study demonstrates that targeted manipulation of cell cycle regulators like p16^INK4A^ represents a promising strategy to enhance the utility of antigen‐specific CD8 T cells for both research and clinical applications. Indeed, recent evidences also demonstrated the role a p16^INK4A^ in the setting of senescence and dysfunctional phenotype in chronically stimulated T cells [21]. Furthermore, *CDKN2A* deletion in CD8 T cells improved proliferation under moderate antigenic stimulation and allowed for extended in vitro culture, crucial for TCR discovery pipeline applications. Additionally, this KO can be useful for ex vivo expansion of TCR‐engineered or CAR T cells isolated from patients, augmenting the number of T cells that can be accumulated for the reinfusion. Of note, *CDKN2A* ablation did not compromise TCR dependency for proliferation, partially addressing safety concerns regarding potential malignant transformation, although further in vivo studies are warranted to confirm biosafety and efficacy. Nevertheless, the frequent association of *CDKN2A* mutations with various cancers [23] necessitates caution regarding its clinical application.

In summary, our findings suggest that knocking out p16^INK4A^ notably enhances the long‐term culture and expansion of antigen‐specific CD8 T cells. This approach holds significant potential for advancing both research and therapeutic applications in adoptive T cell therapies.

## 4 Materials and Methods

### 4.1 Human Blood Samples

Peripheral blood mononuclear cells (PBMCs) were isolated from the blood of four healthy donors who were infected by SARS‐CoV‐2 or vaccinated with SARS‐CoV‐2 Ad‐5 (Oxford–AstraZeneca) or mRNA (Pfizer‐BioNTech)‐based vaccine. Peripheral blood from healthy adult donors was provided by the Department of Transfusion Medicine and Hematology at IRCCS Cà Granda Ospedale Maggiore Policlinico in Milan (708_2020‐IMMUNOM). All the samples were completely anonymized at the source.

### 4.2 Sample Processing, B Cell Isolation, Epstein–Barr Virus (EBV)‐LCL Generation, and Culture

Blood cells were diluted in PBS (Gibco), and PBMCs were isolated by Ficoll‐Paque Plus (GE Healthcare) gradient centrifugation. B cells were isolated from the same donors by positive selection using anti‐CD19 microbeads (Miltenyi Biotech, 130‐050‐301). EBV‐infected‐LCLs were established starting from the isolated B cells: 50 B cells per well were plated in a 384‐well plate and immortalized with virus supernatant containing EBV in the presence of CpG 2006 (Eurofins Genomics) and irradiated allogeneic feeder cells as previously described [24]. Established LCL were cultured in RPMI 1640 medium supplemented with 2 mM glutamine, 1% (vol/vol) nonessential amino acids, 1% (vol/vol) sodium pyruvate, penicillin (50 U/mL), streptomycin (50 µg/mL) (all from Invitrogen), and 10% fetal bovine serum (FBS) (Gibco).

### 4.3 Antigens and Peptides

SARS‐CoV‐2 Ad‐5‐based vaccine (Oxford–AstraZeneca) was kindly gifted by Prof. Andrea Gori (Unit of Infectious Diseases, Foundation IRCCS Ca' Granda Ospedale Maggiore Policlinico, Milan). Synthetic 9 amino acid‐long peptides covering the SARS‐CoV‐2 S protein sequence (UniProtKB: P0DTC2) were produced as crude material on a small scale (2 mg) by Pepscan/Biosynth (Lelystad, the Netherlands).

### 4.4 LCL Transduction With SARS‐CoV‐2 Spike Subunits

A total of 100,000 LCLs of healthy donors were seeded in 96‐well U‐bottom plates in complete RPMI (10% FBS) and transduced with lentiviral vectors (LVVs) provided by Vector Builder (Neu‐Isenburg, Germany) encoding for the subunit S1, or the subunit S2 or the entire Spike protein of SARS‐CoV2 virus. All LVV transductions were done with a multiplicity of infection (MOI) of 5. The day after, the culture medium containing viral particles was replaced with fresh medium. Transduction efficiency was assessed after 7 days at FACS CANTO II by checking the expression of the reporter gene blue fluorescent protein (BFP) included in the LVV. BFP‐positive LCLs were sorted using FACS Aria III SORP (BD Biosciences) to obtain a homogeneous population of LCLs expressing the antigen of interest.

### 4.5 T Cell Culture and Ex Vivo Isolation of Antigen‐Specific CD8 T Cells

T cells were cultured in RPMI 1640 medium supplemented with 2 mM glutamine, 1% (vol/vol) nonessential amino acids, 1% (vol/vol) sodium pyruvate, 0.1% (vol/vol) β‐mercaptoethanol, penicillin (50 U/mL), streptomycin (50 µg/mL) (all from Invitrogen), and 5% human serum (HS) (Swiss donors) with the addition of IL‐2 when indicated (500 IU/mL).

Up to 5 million PBMCs from vaccinated or previously infected donors were labeled with CellTrace Violet Cell (ThermoFisher Scientific, C34557) in 1 mL of PBS at a final concentration of 2.5 µM and incubated for 10 min at 37°C. Cells were then washed twice with complete RPMI (5% HS) and seeded in 96‐well U‐bottom plates at 2 × 10^5^ cells/well in 200 µL of medium. SARS‐CoV‐2 Ad‐5‐based vaccine corresponding to MOI 10 was added to the cells. IL‐2 was added at Day 5 at the final concentration of 50 IU/mL. After 7 days, cells were stained with antibodies anti‐CD25‐PE (clone M‐A251; BD Biosciences; 555432), anti‐CD4‐FITC (clone OKT4, BD Bioscience; 566802), anti‐CD8‐APC (clone B9.11, Beckman Coulter; IM2469), and LIVE/DEAD Fixable Near IR (ThermoFisher, L34980). Proliferating activated T cells were sorted as CTV^low^ CD25^+^ using FACS Aria III (BD Biosciences). Sorted T cells were expanded in vitro in the presence of IL‐2 (500 IU/mL).

### 4.6 Establishment of Spike‐Specific Single–T Cell Clones

T cell clones were isolated from CTV^low^ CD25^+^ antigen‐specific CD8 T cells. After 4–6 days from sorting, antigen‐specific CD8 T cells were cloned by limiting dilution in 384‐well plate at 1 T cells/well in the presence of a mix of irradiated allogeneic PBMCs from 4 donors (25,000 × 10^4^ cells/well), 1 µg/mL of PHA (Remel, ThermoFisher Scientific), and 500 IU/mL of IL‐2. After 14 days, T cells clones were picked and expanded in complete RPMI medium (5% HS) in the presence of 500 IU/mL of IL‐2. T cell clone specificity to SARS‐CoV‐2 Spike was confirmed measuring IFN‐γ and TNF‐α production after overnight co‐culture of T cell clones with WT autologous LCLs or LCLs transduced to express SARS‐CoV‐2 Spike entire protein or S1 or S2 subunits. Briefly, 2 × 10^4^ T cells and 1 × 10^4^ LCLs were seeded in 96‐well U‐bottom plates in complete RPMI medium (5% HS). After 1 h of incubation at 37°C, Brefeldin A (Sigma‐Aldrich, B7651) was added at a final concentration of 10 µg/mL, followed by overnight incubation at 37°C. The day after, cells were washed, stained with anti‐CD8‐APC (clone B9.11, Beckman Coulter; IM2469) and LIVE/DEAD Fixable Near IR (ThermoFisher, L34980), followed by intracellular staining using BD Cytofix/Cytoperm Fixation/Permeabilization Kit with the antibodies anti‐IFN‐γ‐FITC (clone B27, Biolegend, 506504) and anti‐TNFα‐PE (clone MAb11, BD Bioscience, 554513). Stained cells were analyzed at FACS Canto II to identify responder T cell clones.

### 4.7 Assembly of Ribonucleoprotein (RNP) Complexes

Single‐guide RNA sequences (sgRNAs) were designed with the online tool CRISPR Design Tool (Synthego) and synthesized by Synthego (Milan, Italy). RNP complexes were prepared as described [25]. Briefly, sgRNAs were reconstituted to 100 µM in TE buffer supplied by the manufacturer. sgRNA and TrueCut Cas9 Protein v2 (Thermo Fisher Scientific, A36498) were mixed at a ratio of 2.5:1 (40 pmol Cas9 protein per 100 pmol gRNA) and diluted with sterile filtered (0.22 µm) PBS to obtain final concentrations of the RNP complex of 20 µM. The mixture was incubated for 15 min at room temperature and then transiently kept on ice or stored at −80°C until usage.

### 4.8 T Cell Nucleofection

A total of 2 × 10^6^ polyclonal T cells or T cell clones were washed twice with PBS and resuspended in 20 µL P3 Primary Cell Nucleofector Solution buffer prepared with Supplement buffer according to the manufacturer's instructions (P3 Primary Cell 96‐well 4D‐Nucleofector Kit Lonza, V4SP‐3096). For single‐gRNA editing, 2 µL of the 20 µM RNPs were mixed with the cell suspension and transferred into a 16‐well reaction cuvette of the 4D‐Nucleofector System (Lonza). For efficient KO of individual target genes, a mix of two specific, pre‐validated gRNAs was used, 2 µL for each RNP. If three gRNAs were used to target the same gene, 1 µL of each RNP was used. For multiple genes KO up to three genes, 0.5 µL of RNP was used to avoid exceeding 5 µL of total volume of the mix. T cells were nucleofected using the EH100 program of Lonza 4D‐Nucleofector protocol. Immediately after the nucleofection, 100 µL of pre‐warmed RPMI (without supplements) was added to each reaction well, and cells were allowed to recover into the cuvette for 15 min at 37°C. Then the cells were seeded in 96‐well U‐bottom plates at 150,000 cells/well in complete RPMI (5% HS) and 500 IU/mL of IL‐2 and incubated at 37°C, 5% CO_2_. A list of gRNA used in this work is provided in Table S1.

### 4.9 Sanger Sequencing and KO Quantification

One week after nucleofection, edited T cells (5 × 10^4^) were collected, washed twice with PBS, and resuspended in 40 µL of cell lysis buffer made of 1 mM CaCl_2_, 3 mM MgCl_2_, 1 mM EDTA, 1% Triton X‐100, and 10 mM Tris pH 7.5 with the addition of 0.02 mg/mL proteinase K (ThermoFisher Scientific, EO0491). Cells were incubated at 65°C for 10 min, followed by 95°C for 15 min, and then stored at −20°C until usage. A volume of 1 µL of cell lysate was used as the PCR template. The PCR specific for the CRISPR/Cas9 target sites was performed with Dream Taq polymerase (ThermoFisher Scientific DreamTaq PCR Master Mix K1072) using primers targeting the edited locus (see Table S2). The PCR cycler (Thermo Fisher Scientific) settings were 95°C for 5 min, followed by 35 cycles at 95°C for 20 s, 58–65°C (depending on the specific primer pair) for 30 s, and 72°C for 40 s, with the final step at 72°C for 3 min. Sequence amplification was assessed through agarose gel electrophoresis. Successfully amplified fragments were purified using GFX PCR DNA and Gel Band Purification Kits (Cytiva, 28903470) and sequenced by Sanger sequencing performed by Eurofins Genomics. The KO efficiency was analyzed using the Synthego ICE analysis tool (https://ice.synthego.com/) or the TIDE analysis tool (https://tide.nki.nl/).

For the MiSeq benchtop sequencing system (Illumina), a two‐step PCR barcoding scheme was performed. A volume of 1 µL of cell lysate was used to perform PCR‐I with primers targeting the edited locus and carrying Illumina sequencing barcodes (Table S2). The MiSeq PCR reactions were carried out with 0.25 µL of Phusion High‐Fidelity DNA Polymerase (ThermoFisher Scientific, F530L), 5 µL 5× high‐fidelity PCR buffer (ThermoFisher Scientific), 0.5 µL dNTPs (10 mM stock; Thermo Fisher Scientific), 1.2 µL forward primer (10 µM stock), 1.2 µL reverse primer (10 µM stock), 1 µL lysate, and 15.85 µL H_2_O. A volume of 1–2 µL of PCR‐I was used to perform PCR‐II using the same cycling conditions and a combination of barcode primers that is unique for each clone to be analyzed as previously described [26]. The PCR reaction was run on a 1% agarose gel, purified, and sequenced by Illumina MiSeq sequencing. The results of the MiSeq were analyzed with the Outknocker 2.0 webtool (http://www.outknocker.org/outknocker2.htm).

### 4.10 Killing Assay

Target autologous LCLs were stained with CFSE (Invitrogen, C34570) at 0.5 µM in PBS for 10 min at 37°C with a cell density of 5 million/mL to distinguish them from the CD8 effector cells. After 3 steps of washing with complete RPMI medium (5% HS) without the addition of IL‐2, target cells were co‐cultured with effector CD8 T cell clones in 96‐well U‐bottom plates at 1:1 effector to target ratios in the presence of titrated concentrations of the peptide recognized by the CD8 T cell clone (starting concentration 1 µg/mL). In alternative, CFSE‐stained autologous LCLs expressing Spike subunits were co‐cultured with CD8 T cell clones in 96‐well U‐bottom plates at different effector‐to‐target ratios. After 6 h or overnight incubation, cells were washed, stained with LIVE/DEAD Fixable Near‐IR (Invitrogen, L10119), and acquired at FACS CANTO II at fixed acquisition time. LCLs cultured without effector cells were used as a control for 0% killing. The specific killing was calculated as


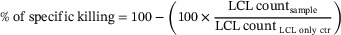




### 4.11 Cytokines Quantification

CD8 T cell clones were co‐cultured with autologous LCL pulsed with different concentration of the antigenic peptide in complete T cell medium. After overnight incubation, the supernatant was recovered for cytokine release studies. INF‐γ release was measured by ELISA assay using BD OptEIA Human IFN‐γ ELISA Set (BD Bioscience, 555142). TNF‐α, IL‐2, FasL, Granzyme A, Granzyme B, and Perforin‐1 release were measured by xMAP INTELLIFLEX System‐Luminex.

### 4.12 T Cell Count and Cell Trace CFSE Proliferation Assay

Absolute CD8 T cell count was performed by flow cytometry using CountBright Plus Absolute Counting Beads (Invitrogen, C36995). Briefly, an aliquot of T cells was mixed with 5 µL of counting beads and 45 µL of FACS buffer (PBS, 1% FBS, and 5 mM EDTA) and immediately acquired at FACS Canto II at fixed acquisition rate. For proliferation assay, CD8 T cell clones were stained with CFSE (Invitrogen, C34570) at 0.5 µM in PBS for 10 min at 37°C. Then, T cells were washed three times with complete RPMI medium without the addition of IL‐2 and cultured 96‐well U‐bottom plates in the presence of different ratios of irradiated autologous LCLs expressing Spike subunit or in the presence of homeostatic cytokines. Recombinant cytokines were used at concentrations of 25 ng/mL (IL‐7, IL‐15; Peprotech), 10 ng/mL (IL‐6, IL‐10; Peprotech), and 1000 IU/mL of IL‐2 unless otherwise indicated. After 7 days of culture, T cells were washed, stained with anti‐CD3‐APC (OKT3 clone, Biolegend, 317318) and LIVE/DEAD Fixable Near‐IR (Invitrogen, L10119), and acquired at FACS CANTO II at fixed time of acquisition.

### 4.13 Repetitive Stimulation Assay

CD8 T cell clones and target autologous LCLs expressing S1 subunit were seeded in 96‐well U bottom plate in multiple wells at T‐to‐LCL‐ratio of 1:1 in complete T cell medium with the addition of IL‐2 (50 IU/mL). Fresh LCLs‐S1 were added at a fixed initial ratio of 1:1 (LCL:T ratio) every 48 h. After 24 h from each LCL addition, cells from one well were stained with LIVE/DEAD Fixable Near‐IR (Invitrogen, L10119), anti‐CD19 Pe‐Cy7 (clone SJ25C1, BD Pharmingen; 557835), and anti‐CD8 (clone B9.11, Beckman Coulter; IM2469) and subsequently acquired at FACS Canto II (BD Bioscience).

### 4.14 Flow Cytometry

CD8 T cell clones were kept resting or activated in co‐culture with autologous LCLs expressing Spike subunit or WT LCLs pulsed with cognate peptide and were analyzed at different time points for surface markers using the following antibodies: anti‐CD3‐APC (clone OKT3, Biolegend; 317318), anti‐CD8‐APC (clone B9.11, Beckman Coulter; IM2469), anti‐CD25‐PE (clone M‐A251, BD Bioscience; 555432), or anti‐CD25‐Alexa Fluor 647 (clone BC96, Biolegend; 302618), anti‐CD38‐FITC (clone T16, Beckman Coulter; A07778). For intracellular staining, cells were fixed and permeabilized with Cytofix/Cytoperm (BD Biosciences, 554722) and then stained with the following antibodies: anti‐Granzyme A‐PE Cy7 (clone CB9, Biolegend, 507221), anti‐Granzyme B‐FITC (clone GB11, Biolegend, 515403), and anti‐Perforin‐BV421 (clone dG9, Biolegend; 308122). Stained cells were resuspended in FACS buffer (PBS, 1% FBS, and 5 mM EDTA) and acquired at FACS Canto II (BD Bioscience). To obtain the absolute T cell count of cells analyzed, 5 µL of CountBright Plus Absolute Counting Beads (Invitrogen, C36995) were added to stained T cells and then acquired at a fixed acquisition rate.

### 4.15 Single‐Cell RNA‐Seq Library Preparation and Sequencing

Total CD8 T cells for single‐cell RNA‐seq were isolated from PBMCs of one healthy donor by FACS sorting. PBMCs were stained with anti‐CD3‐BUV, anti‐CD8‐APC (clone B9.11, Beckman Coulter; IM2469), anti‐CD19‐Pe‐Cy7, anti‐CD4‐FITC (clone OKT4, BD Bioscience; 566802), and anti‐CD14 PeCy5, and total CD8 T cells were sorted as CD3^+^/CD8^+^/CD4^−^/CD19^−^/CD14^−^. TCR and gene expression libraries were generated using Chromium Next GEM Single Cell V(D)J Reagent Kit v1.1 (10X Genomics) according to the manufacturer's instructions. Sorted T cell populations were used to generate paired heavy and light chain TCR libraries. Up to 20,000 cells were loaded in the 10X Genomics Chromium Controller to generate single‐cell gel‐beads in emulsion. After reverse transcription, gel‐beads in emulsion were disrupted. Barcoded cDNA was isolated and used for the preparation of TCR and gene expression libraries. Each step was conducted as specified in the 10X Genomics user guide kit v.2. The purified libraries were sequenced with NovaSeq (Illumina) following the instructions provided in 10X Genomics user guide for the read length and depth.

### 4.16 Single‐Cell RNA‐Seq Data Analysis, Processing, Annotation, and Differential Gene Expression Analysis

Preprocessing of the raw sequencing data involved the utilization of the 10X Genomics Cell Ranger pipeline (version 7.2.0). Cellranger mkfastq was used to demultiplex the libraries, segregating them based on sample indices and converting the barcode and read data into FASTQ files. Cellranger multi was then employed to process the FASTQ files and align them to the human GRCh38 reference genome. To aggregate the libraries, Cellranger aggr was used. Quality control was performed to remove poor‐quality cells and badly detected genes. For transcriptome analysis, we used the CellRanger pipeline 9.0.1 and Seurat package 5.3 for quantification, quality control, data normalization, dimensionality reduction, clustering, differential expression analysis, and data visualization. We used the CellRanger pipeline to generate gene expression count matrices from the raw data. For each sample, a gene‐by‐cell counts matrix was used to create a Seurat object using Seurat5.3. We filtered cell barcodes with >200 and <6000 UMIs as well as <15% mitochondrial contents and >10% ribosomal content. The sample was then normalized by a factor of 10,000 and log transformed (NormalizeData). The top 6000 most variable genes were then identified using the FindVariableFeatures method. The gene expression matrix obtained by applying the filtering steps above was then used to perform principal‐component analysis (RunPCA); preliminary clustering analysis, including nearest neighbor graph (FindNeighbors) and unbiased clustering (FindClusters); and cell type annotation. UMAP was then used to visualize the expression data. We identified gene expression markers for each cluster using FindAllMarkers from Seurat with default settings, including Wilcoxon test and Bonferroni *p* value correction. Differential gene expression between specified clusters (or subclusters) was performed using FindMarkers (Wilcoxon rank‐sum test) with Benjamini–Hochberg false discovery rate (FDR) correction and average log fold change (FC). Genes were considered (significantly) differentially expressed if FDR < 0.05 and log FC > 0.2 in a given group. All computational analyses were performed in R (v.4.4.1). Single‐cell RNA sequencing data were deposited on the ArrayExpress collection in BioStudies (Accession number E‐MTAB‐15677).

## Author Contributions

**Silvia Fiori**: methodology, investigation, visualization, discussion, writing of original draft, review and editing. **Cecilia Adragna**: methodology, reagents, review, and editing. **Emilia Malvicini**: methodology, review, and editing. **Tommaso Basini**: methodology, review, and editing. **Donatella Galgano**: reagents, review, and editing. **Edoardo Scarpa**: review and editing. **Sandra Jovic**: methodology, review, and editing. **Niklas A. Schmacke**: methodology, review, and editing. **Veit Hornung**: reagents, review, and editing. **Federica Sallusto**: conceptualization, review, and editing. **Ludovica Bruno**: investigation, review, and editing. **Antonio Lanzavecchia**: conceptualization, investigation, supervision, discussion, writing of original draft, review, and editing. **Manuel Albanese**: conceptualization, methodology, investigation, visualization, supervision, discussion, writing of original draft, review, and editing.

## Conflicts of Interest

The authors declare no conflicts of interest.

## Supporting information




**Supporting File**: eji70224‐sup‐0001‐SuppMat.pdf.

## Data Availability

The data that support the findings of this study are available in the Supporting Information section of this article. Single‐cell RNA sequencing data that support the findings of this study are openly available in the ArrayExpress collection in BioStudies at https://www.ebi.ac.uk/biostudies/arrayexpress, reference number [E‐MTAB‐15677]. All further relevant source data that support the findings of this study are available from the corresponding authors upon reasonable request.
