# Supplementary material for: CRISPR/Cas9‐Mediated Gene Knockout Reveals a Nonredundant Role for p16INK4A in Controlling TCR‐Dependent and Independent CD8 T Cell Expansion
Source: Eur J Immunol. 2026 Jun 25;56(6):e70224. doi: 10.1002/eji.70224 (PMC13296263; doi:10.1002/eji.70224)
Supplement: Supplementary file 1 — Supporting File: eji70224‐sup‐0001‐SuppMat.pdf. [file EJI-56-e70224-s001.pdf]

Supplementary information

CRISPR/Cas9-mediated gene knockout reveals a non-redundant role for p16^{INK4A} in controlling TCR-dependent and independent CD8 T cell expansion

Silvia Fiori, Cecilia Adragna, Emilia Malvicini, Tommaso Basini, Donatella Galgano, Edoardo Scarpa, Sandra Jovic, Niklas A. Schmacke, Veit Hornung, Federica Sallusto, Ludovica Bruno, Antonio Lanzavecchia, and Manuel Albanese

Supplementary Figure 1

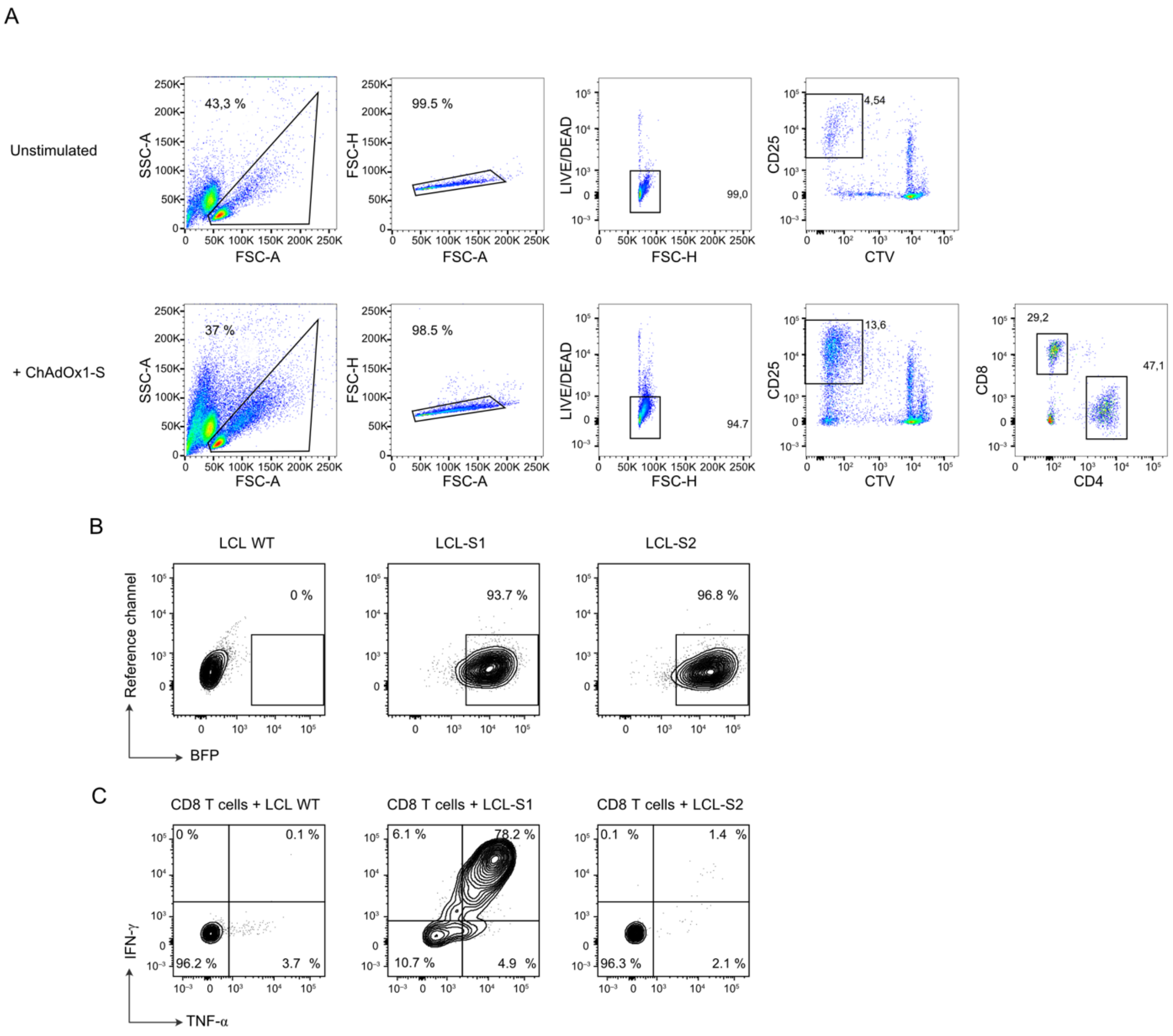

Figure S1. Generation of antigen specific CD8 T cell clones.
(**A**) Sorting strategy of activated and proliferating CD8 T cells, defined as CD25+ and CTV^{low}, from total PBMCs. First line shows unstimulated control. Second line shows PBMCs stimulated with SARS-CoV-2 Ad-5 based vaccine (ChAdOx1-S1 Oxford–AstraZeneca). (**B**) Expression of Blue Fluorescent Protein (BFP) marker in LCL transduced with a lentiviral vector expressing the SARS-CoV-2 Spike subunits S1 or S2 linked via a T2A sequence to a BFP marker. (**C**) Specificity of CD8 T cell clones assessed as TNF-α and IFN-γ production measured by flow cytometry upon overnight co-culture of T cells with autologous LCL expressing Spike S1 or S2 subunits of SARS-CoV-2. One representative S1-specific clone is shown.

Supplementary Figure 2

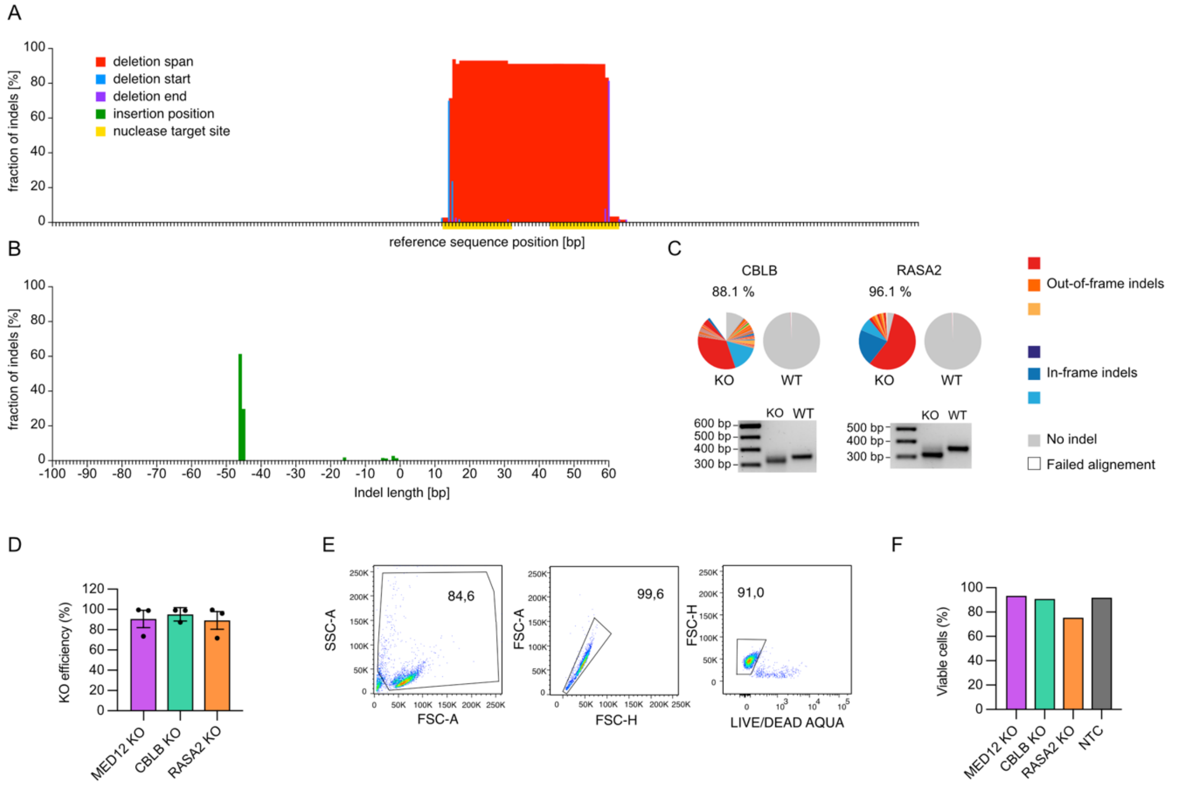

Figure S2. Gene editing efficiency evaluation.
Analysis of position (**A**) and size distribution (**B**) of the indels from RASA2 KO sample generated with 2 gRNAs spanning 46 nt from each other. One representative experiment is shown (n=3). Outknocker (http://www.outknocker.org/) was used for sequence analysis. (**C**) Knockout efficiency at CBLB and RASA2 gene locus detected by Illumina MiSeq sequencing and gel electrophoresis of the corresponding PCR of the target genomic locus. Representative pie charts and gel electrophoresis showing PCR results performed on the target locus. One experiment for each target gene is shown (n=3). (**D**) Quantification of knockout efficiency shown in (**C**). (**E**) Gating strategy for T cell viability assessment. CD8 T cell clones with different knockouts were cultured for 10-15 days, with half medium changes to refresh IL-2 and then viability was assessed by flow cytometry using Live/Dead Aqua staining. (**F**) Bar plot showing viability of NTC, MED12 KO, CBL-B KO, and RASA2 KO CD8 T cell clones measured after 10-15 days from knockout.

Supplementary Figure 3

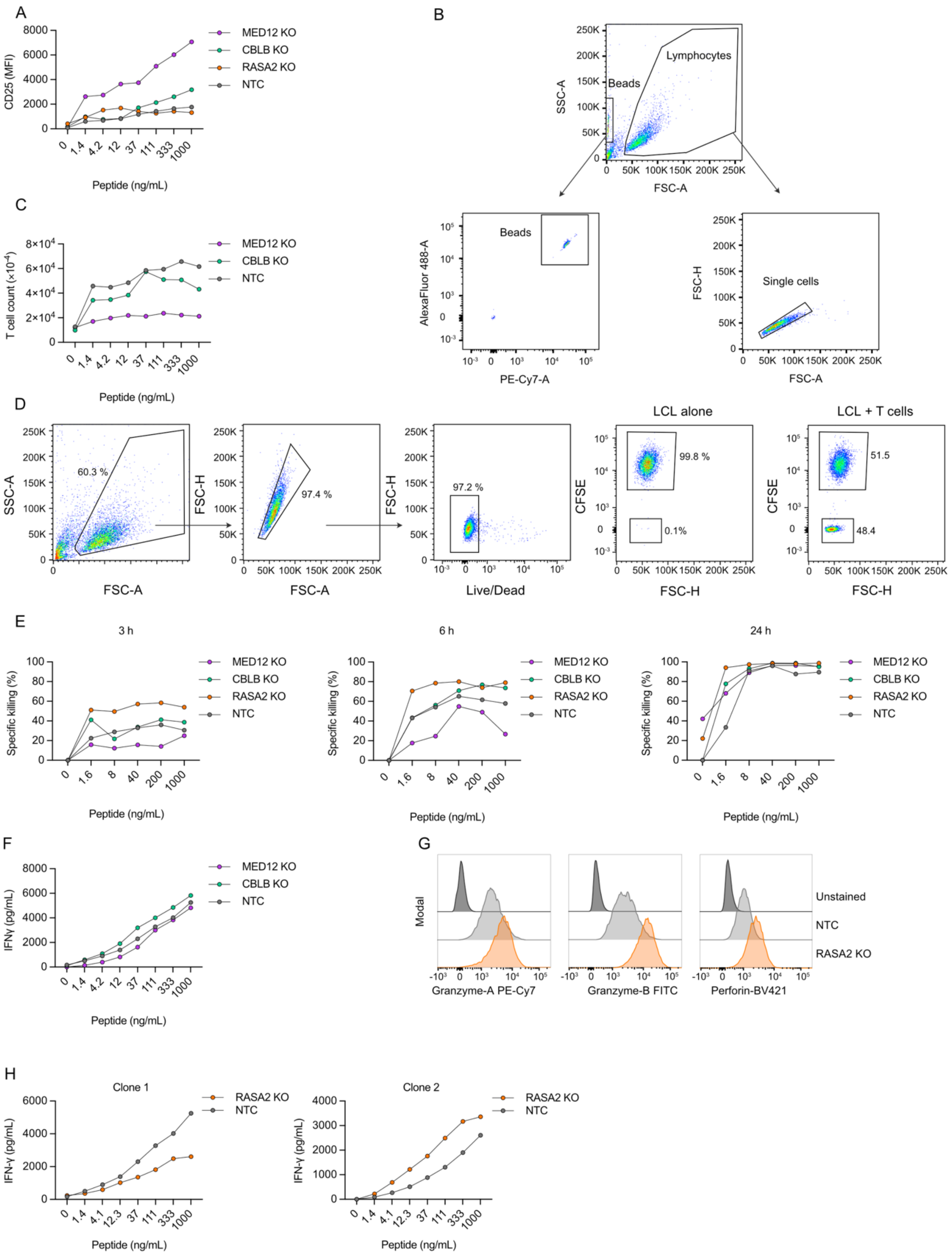

Figure S3. Characterization of effector functions of MED12 KO, CBL-B KO and RASA2 KO CD8 T cell clones.

(**A**) Surface expression of CD25 on MED12 KO, CBL-B KO, RASA2 KO and NTC SARS-CoV-2 Spike-specific CD8 T cell clones in response to autologous LCL pulsed with different concentrations of cognate peptide. One clone from one donor is represented (n=1). (**B**) Gating strategy for absolute T cell count with fluorescent counting beads, used in Fig. 1E, 2F and S3C. Equal amounts of beads were added in the samples in FACS buffer before acquisition at flow cytometry. (**C**) Proliferation measured as absolute T cell count of a NTC, MED12 KO and CBL-B KO SARS-CoV-2 Spike-specific CD8 T cell clones in response to autologous LCLs pulsed with different concentrations of Spike peptides. T:LCL ratio used was 2:1. Cell count was measured by flow cytometry 5 days after stimulation. (n=1). (**D**) Gating strategy for flow cytometry based killing assay. Target cells (LCLs) were labelled with CFSE. (**E**) CD8-mediated cytotoxicity of NTC, MED12 KO, CBL-B KO and RASA2 KO SARS-CoV-2 Spike-specific CD8 T cell clones measured as percentage of the specific killing of autologous LCLs in the presence of different concentrations of cognate Spike peptide. T:LCL ratio used was 1:1. LCLs killing was measured by flow cytometry after 3 hours, 6 hours and 24 hours. Results from one donor are shown (n=1). (**F**) IFN-γ production measured by ELISA of NTC, MED12 KO, CBLB KO Spike-specific CD8 T cell clone upon overnight co-coculture with autologous LCLs pulsed with titrated concentration of Spike cognate peptide. Result from one donor are shown (n=1). (**G**) Representative histograms of intracellular expression of GZMA, GZMB, and Perforin-1 measured by flow cytometry in RASA2 KO and NTC CD8 T cell clones quantified in Fig. 1D. (**H**) IFN-γ production measured as in Fig. S3G of NTC and RASA2 KO Spike-specific CD8 T cell clones. Results from two clones from two donors are shown (n=2).

Supplementary Figure 4

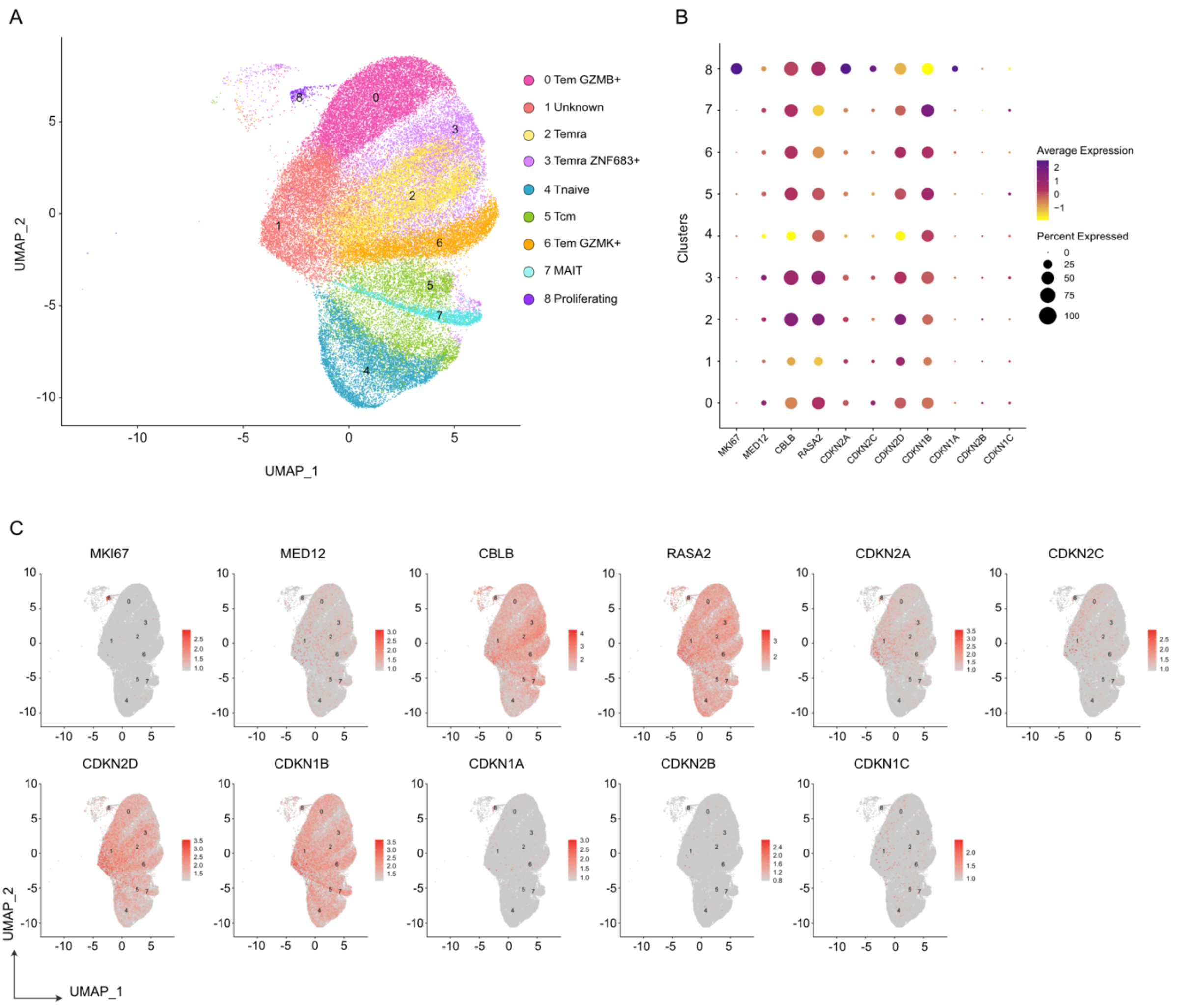

Figure S4. Differential expression of knockout targeted genes in CD8 T cell subsets
(A) Two-dimensional UMAP projection of single-cell gene expression data of circulating total CD8 T cells from one healthy donor. (**B, C**) Dot plot (**B**) and UMAP (**C**) representing the expression levels of MKI67 gene and the genes selected for knockout within the different UMAP clusters. Note the different Log2 expression levels among the selected genes.

Supplementary Figure 5

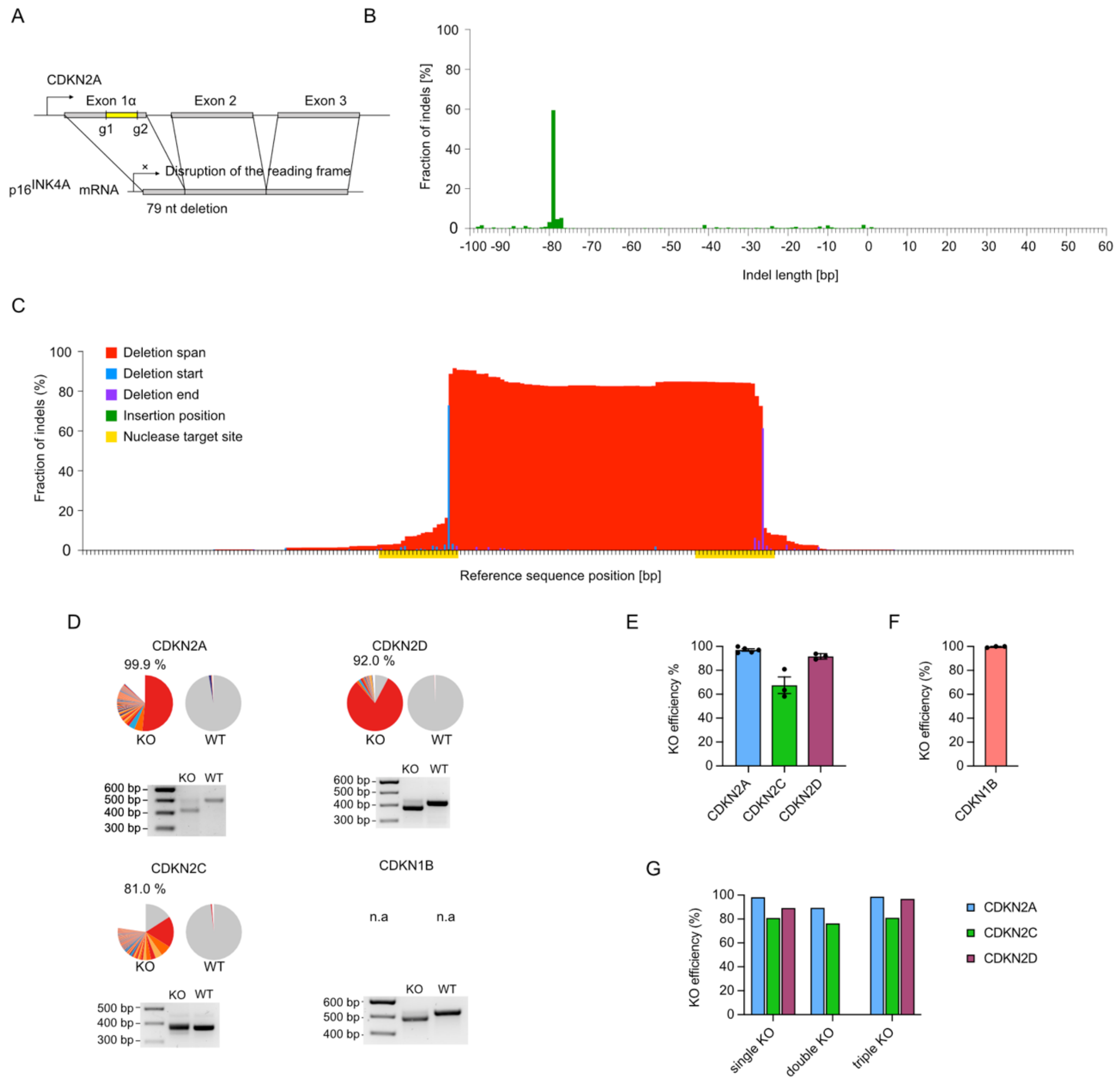

Figure S5. Evaluation of gene editing efficiency of CDKi inhibitor knockouts

(**A**) Representative scheme of knockout in CDKN2A locus using 2 gRNAs. (**B-C**) Analysis of the size distribution (**B**) and position (**C**) of the indels from CDKN2A KO generated with 2 gRNA spanning 79 nt from each other. One representative experiment is shown (n=6). Outknocker (http://www.outknocker.org/) was used for sequence analysis. (**D**) Knockout efficiency at CDKN2A, CDKN2D, CDKN2C and CDKN1B genes detected by Illumina MiSeq sequencing and gel electrophoresis of the corresponding PCR of the target genomic locus. Representative pie charts and gel electrophoresis showing PCR results performed on the target locus for each target gene. One representative experiment is shown (n=3). (**E**) Quantification of knockout efficiencies shown in (**D**). (**F**) Quantification of knockout efficiency at CDKN1B locus, detected by Sanger sequencing and ICE analysis (https://ice.editco.bio/#/). (**G**) Quantification of knockout efficiencies of single, double and triple knockout of CDKN2A, CDKN2D and CDKN2C genes, measured by Illumina Miseq sequencing and outknocker analysis. (n=1).

Supplementary Figure 6

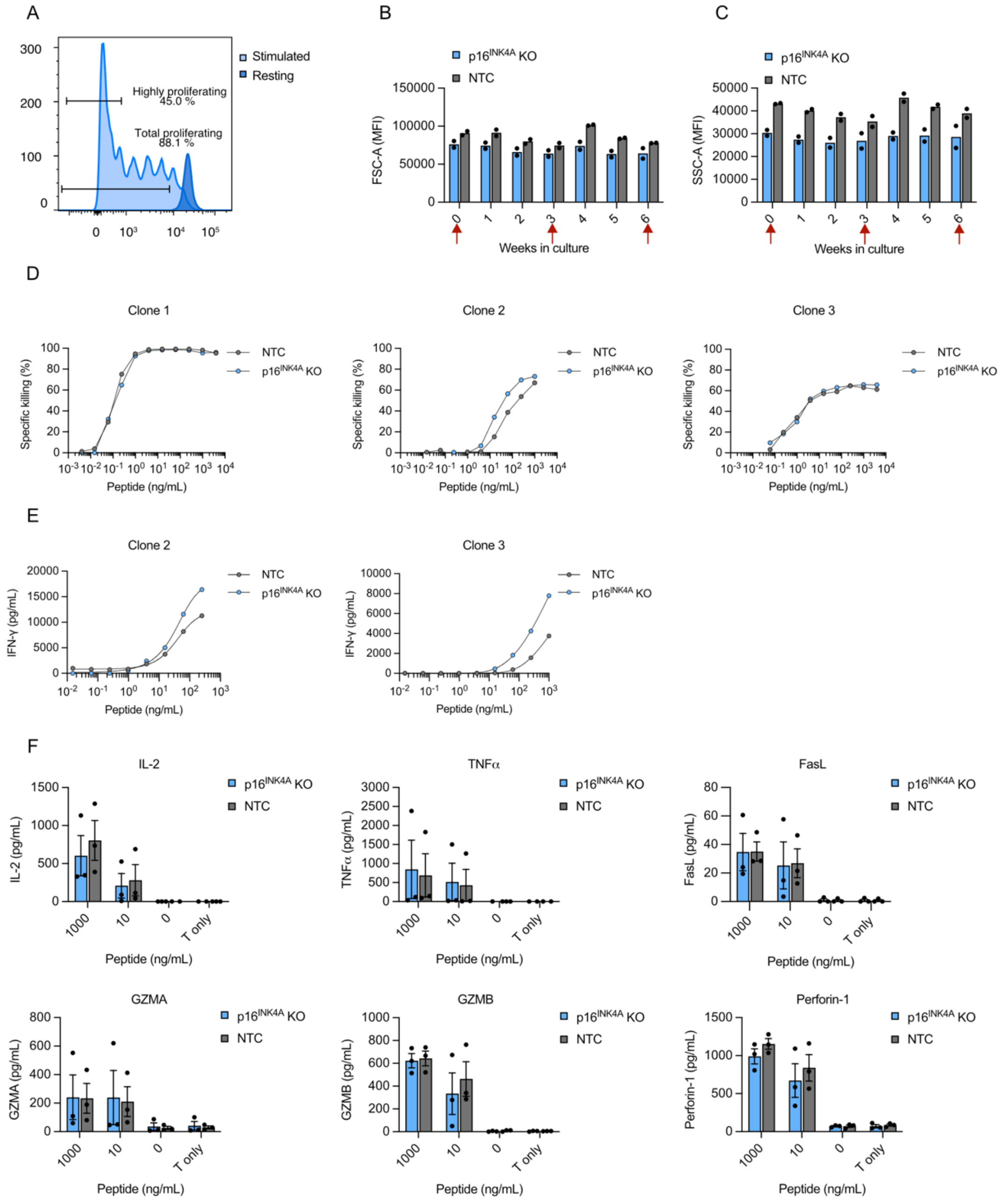

Figure S6. Phenotype characterization of p16^{INK4A} knockout antigen-specific CD8 T cell clones. (**A**) Gating strategy for quantification of proliferation by CFSE dilution, used in Fig. 2 B, D and E. (**B, C**) Size and granularity of NTC and p16^{INK4A} KO SARS-CoV-2 Spike-specific CD8 T cell clones measured as FSC-A and SSC-A by flow cytometry. T cells were restimulated with PHA and allogeneic irradiated feeders every 3 weeks (time points indicated with red arrows in the x axis). Measurements were performed by flow cytometry every week. Results from 2 donors are shown (n=2). (**D**) CD8 mediated cytotoxicity of Spike-specific CD8 T cell clone measured as percentage of specific killing of autologous LCLs in the presence of different concentrations of cognate peptide. T:LCL ratio used was 1:1. Percentage of LCL killing was measured by flow cytometry after 24 hours. Results from three donors are shown. (n=3). (**E**) IFN-γ production measured by ELISA of NTC and p16^{INK4A} KO Spike-specific CD8 T cell clone upon overnight co-coculture with autologous LCLs pulsed with titrated concentration of Spike cognate peptides. Two representative experiments out of 3 different donors are shown (n=3). (**F**) Overnight production of IL-2, TNF-α, Fas Ligand (FasL), GZMA, GZMB and Perforin-1 of NTC and p16^{INK4A} KO of Spike-specific CD8 T cell clones detected in the supernatant by Luminex assay. Cells were stimulated with autologous LCLs in the presence of different concentrations of the cognate peptide. Results from three donors are shown. Mean and s.e.m are shown (n=3). Paired t test was performed resulting in no statistical significance.

Supplementary Figure 7

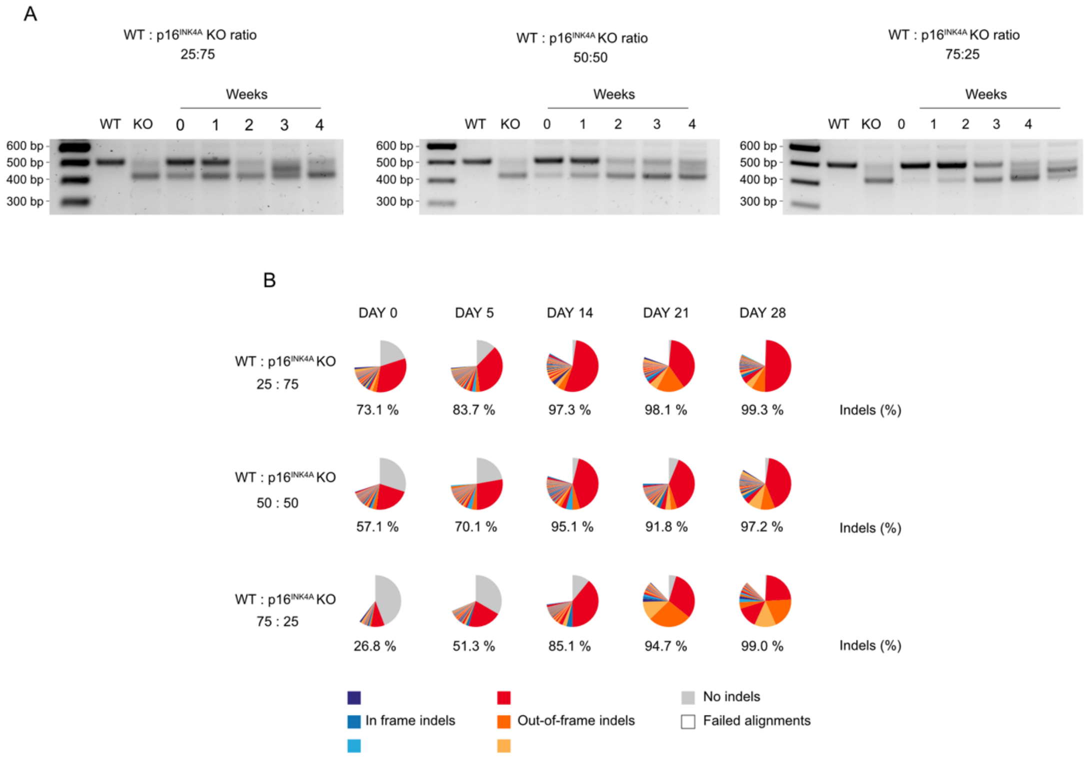

Figure S7. Competitive proliferation experiment between WT and p16^{INK4A} KO CD8 T cell clone.
(**A**) Gel electrophoresis of PCR on CDKN2A locus obtained in the proliferation competition experiment between WT and p16^{INK4A} KO CD8 T cell clone (Fig. 4 C, D). The experiment was performed mixing WT and p16^{INK4A} KO clone in the ratio of 25:75, 50:50, and 75:25 (WT:KO) and then stimulating T cells with autologous LCL expressing Spike-S1 subunit. Cell lysate was acquired in the following weeks and PCR on the target locus was performed. (**B**) Pie chart showing the frequency of Indels at each time point, quantified in figure 4D.

Supplementary Table 1: List of gRNA used

gRNA name	gene target	sequence	PAM	strand	comment
RASA2-gRNA1	RASA2	AUUUUGUGGGGUCCAAGAUA	TGG	-	Carnevale et al, Nature, 2022
RASA2-gRNA2	RASA2	CUGUACCAUAAAUUUGGACC	AGG	+	
CBLB-gRNA1	CBLB	AAAUAUCAAGUAUAUAUGGU	GGG	-	Carnevale et al, Nature, 2022
CBLB-gRNA2	CBLB	CGUAAAUGCUGAUAUGUAUC	AGG	-	
MED12-gRNA1	MED12	ACGGCCUUGAAUGUAAAACA	AGG	+	
MED12-gRNA2	MED12	UAACCAGCCUGCUGUCUCUG	GGG	+	Freitas et al, Science, 2022
p16-gRNA1	CDKN2A	UUCGGCUGACUGGCUGGCCA	CGG	+	
p16-gRNA2	CDKN2A	CCCAACGCACCGAATAGTTA	CGG	+	Albanese et al, Plos pathogens 2021
p18-gRNA1	CDKN2C	CCUAAAGAAUGGCCGAGCCU	TGG	+	
p18-gRNA2	CDKN2C	CGAGCCUUGGGGGAACGAGU	TGG	+	
p18-gRNA3	CDKN2C	UUGGCGUCCGCAGCUGCCAG	GGG	+	
p19-gRNA1	CDKN2D	CAGCAGCUCCAGGGCGAUGG	CGG	-	
p19-gRNA2	CDKN2D	CGGAGGUGUCCUGGACAUUG	GGG	-	
p27-gRNA1	CDKN1B	ACCCCAAGCCCUCGGCCUGC	AGG	+	
p27-gRNA2	CDKN1B	CUGCAGGAACCUCUUCGGCC	CGG	+	
p27-gRNA3	CDKN1B	AGUCCCGGGUUAACUCUUCG	TGG	-	
NTC synthego	Non targeting control	GCACUACCAGAGCUAACUCA			

Supplementary Table 2: List of primers used for PCR reactions.

Gene target	Strand	Sequence	Used for
RASA2	For	gttctttcagattgaagtcatcc	Sanger Sequencing / TIDE analysis
RASA2	Rev	aggaaagttcaaacgaataggc	Sanger Sequencing / TIDE analysis
MED12	For	tgttctacacggaaccctcc	Sanger Sequencing / TIDE analysis
MED12	Rev	gaagaaggggcagacaggtg	Sanger Sequencing / TIDE analysis
CBLB	For	gaatggtaggagaacagaatagg	Sanger Sequencing / TIDE analysis
CBLB	rev	caaagcgactcacatttaccc	Sanger Sequencing / TIDE analysis
CBLB	rev	gtgcggtaggtaataccatc	Sanger Sequencing / TIDE analysis
CDKN2A	For	aaaactccccaggaagcctc	Sanger Sequencing / TIDE analysis
CDKN2A	Rev	taatagcacctcctccgagc	Sanger Sequencing / TIDE analysis
CDKN2C	For	ccaatggctcagttttgctg	Sanger Sequencing / TIDE analysis
CDKN2C	Rev	gacttgttttccccacctctc	Sanger Sequencing / TIDE analysis
CDKN2D	For	acccctcctctagcatttcc	Sanger Sequencing / TIDE analysis
CDKN2D	Rev	acagcagtgtgaccctcttg	Sanger Sequencing / TIDE analysis
CDKN1B	For	tttttgagagtgcgagagagg	Sanger Sequencing / TIDE analysis
CDKN1B	Rev	tgcgtgtcctcagagttagc	Sanger Sequencing / TIDE analysis
CDKN2A	For	acactctttccctacacgacgctcttccgatctctgcggagagggggagag	Illumina Miseq System / Gene editing validation
CDKN2A	Rev	tgactggagttcagacgtgtgctcttccgatctcgtcctccagagtcgccc	Illumina Miseq System / Gene editing validation
CDKN2C	For	tgactggagttcagacgtgtgctcttccgatctccaatggctcagttttgctg	Illumina Miseq System / Gene editing validation
CDKN2C	Rev	acactctttccctacacgacgctcttccgatctgacttgttttccccacctctc	Illumina Miseq System / Gene editing validation
CDKN2D	For	acactctttccctacacgacgctcttccgatctacccctcctctagcatttcc	Illumina Miseq System / Gene editing validation
CDKN2D	Rev	tgactggagttcagacgtgtgctcttccgatctacagcagtgtgaccctcttg	Illumina Miseq System / Gene editing validation
CDKN1B	For	acactctttccctacacgacgctcttccgatctttttttgagagtgcgagagagg	Illumina Miseq System / Gene editing validation
CDKN1B	Rev	tgactggagttcagacgtgtgctcttccgatcttgcgtgtcctcagagttagc	Illumina Miseq System / Gene editing validation

RASA2	For	acactctttccctacacgacgctcttccgatctgttctttcagattgaagtcatcc	Illumina Miseq System / Gene editing validation
RASA2	Rev	tgactggagttcagacgtgtgctcttccgatctactgtttctgtcttgtcttcttg	Illumina Miseq System / Gene editing validation
RASA2	For	acactctttccctacacgacgctcttccgatcttaaggcttagtatgaagcagttg	Illumina Miseq System / Gene editing validation
RASA2	Rev	tgactggagttcagacgtgtgctcttccgatctattttgaggcaatgaaaagcc	Illumina Miseq System / Gene editing validation
CBLB	For	tgactggagttcagacgtgtgctcttccgatctgaagaaataggacatggttctc	Illumina Miseq System / Gene editing validation
CBLB	Rev	acactctttccctacacgacgctcttccgatctagtcttattgcccgttttgac	Illumina Miseq System / Gene editing validation
MED12	For	acactctttccctacacgacgctcttccgatcttgttctacacggaaccctcc	Illumina Miseq System / Gene editing validation
MED12	Rev	tgactggagttcagacgtgtgctcttccgatctgaagaaggggcagacaggtg	Illumina Miseq System / Gene editing validation
